# Evaluation of a method to measure HHV-6B infection in vitro based on cell size

**DOI:** 10.1186/s12985-017-0917-z

**Published:** 2018-01-05

**Authors:** Aniuska Becerra-Artiles, Tessa Santoro, Lawrence J. Stern

**Affiliations:** 1 Department of Pathology, University of Massachusetts Medical School, 55 Lake Ave North, Worcester, MA 01655 USA; 2 Department of Pathology, Department of Biochemistry and Molecular Pharmacology, University of Massachusetts Medical School, 55 Lake Ave North, Worcester, MA 01655 USA

**Keywords:** HHV-6B, Cytopathic effect, Cell size, Imaging-based automated cell counter

## Abstract

**Background:**

Human herpesvirus 6 (HHV-6A and HHV-6B) infection of cell cultures can be measured by different methods, including immunofluorescence microscopy, flow cytometry, or quantification of virus DNA by qPCR. These methods are reliable and sensitive but require long processing times and can be costly. Another method used in the field relies on the identification of enlarged cells in the culture; this method requires little sample processing and is relatively fast. However, visual inspection of cell cultures can be subjective and it can be difficult to establish clear criteria to decide if a cell is enlarged. To overcome these issues, we explored a method to monitor HHV-6B infections based on the systematic and objective measurement of the size of cells using an imaging-based automated cell counter.

**Results:**

The size of cells in non-infected and HHV-6B-infected cultures was measured at different times post-infection. The relatively narrow size distribution observed for non-infected cultures contrasted with the broader distributions observed in infected cultures. The average size of cultures shifted towards higher values after infection, and the differences were significant for cultures infected with relatively high doses of virus and/or screened at longer times post-infection. Correlation analysis showed that the trend observed for average size was similar to the trend observed for two other methods to measure infection: amount of virus DNA in supernatant and the percentage of cells expressing a viral antigen. In order to determine the performance of the size-based method in differentiating non-infected and infected cells, receiver operating characteristic (ROC) curves were used to analyze the data. Analysis using size of individual cells showed a moderate performance in detecting infected cells (area under the curve (AUC) ~ 0.80-0.87), while analysis using the average size of cells showed a very good performance in detecting infected cultures (AUC ~ 0.99).

**Conclusions:**

The size-based method proved to be useful in monitoring HHV-6B infections for cultures where a substantial fraction of cells were infected and when monitored at longer times post-infection, with the advantage of being relatively fast and easy. It is a convenient method for monitoring virus production in-vitro and bulk infection of cells.

**Electronic supplementary material:**

The online version of this article (10.1186/s12985-017-0917-z) contains supplementary material, which is available to authorized users.

## Background

Since the discovery of the human herpesvirus 6A (HHV-6A) and HHV-6B, a characteristic cytopathic effect induced in cells has been described. For instance, infected cells are characterized by the occurrence of enlarged cells, first described as “balloon-like” syncytia [1]. Presence of balloon-like cells has often been used as an indicator of HHV-6 infection of primary cells cultures, ex-vivo infection of primary cells, and in-vitro infection of various cell lines [1–4]. However, visual inspection of cell cultures is subjective and it can be difficult to establish clear criteria to decide if a cell is infected. Quantitative or semi-quantitative methods widely used to determine HHV-6 infection include immunofluorescence microscopy (IFA), flow cytometry, and molecular methods. In IFA, antibodies specific for HHV-6 proteins allow the identification of infected cells; this method has been used since early days of HHV-6 research [2, 3]. HHV-6 antisera or monoclonal antibodies to various virus proteins have been used, including antibodies recognizing glycoproteins B (gB), gH, gQ, and protein U27 among others [5–8]. A shortcoming of this method is that it requires a lengthy sample processing with multiple incubation and washing steps, as well as the scanning of randomized fields in an adequate microscope to obtain a representative sample adequate for quantitative analysis. In addition, IFA-based methods are subjected to person-to-person variability in the read-out, often requiring readings by two or more experienced investigators. An alternative is the analysis of cells stained with the fluorescent-labeled antibodies by flow cytometry [9–11]. This method circumvents the subjectivity of the microscopy methods, although still requires multiple incubations and washing steps and adequate instrumentation. Molecular methods to detect HHV-6 infection include detection of expression of viral genes by reverse-transcription PCR [11–13] and measurement of the amount of viral genome in infected cells or cell culture supernatants by quantitative PCR. Quantitative-PCR (qPCR)-based methods are very sensitive and are widely used [14–18]. However, they require specialized equipment, reagents, and sample processing, and do not provide information about the actual number or frequency of infected cells or the amount of infectious virus produced, and so represent only an indirect measure of the infection.

The search of a fast and simple method to objectively monitor HHV-6 infection in-vitro on a daily basis led us to explore the use of an imaging-based automated cell counter able to measure the size of cells in a cell suspension. Measurements of the average size of cells (usually as cell diameter) using automated cell counters have been used for some time to monitor infections, in particular in cell culture systems set up for recombinant protein production using insect cells. For example, infection of Sf-9 cells with Baculovirus expression vectors results in changes in average size during the progress of infection, and monitoring the average size of the cells has been useful to estimate the degree of infection [19], to predict the yield of recombinant protein produced [20], and also to estimate virus titers [21]. The work presented in this paper consists of a series of analyses that explore the feasibility of using a cell counter to routinely monitor HHV-6B infections in SupT1 cells. Results suggest that this method is fairly reliable to estimate infections when high doses of virus and/or long times are used; when compared to other methods like IFA, flow cytometry and qPCR, it is faster and simpler.

## Methods

### Virus

HHV-6B strain Z29 was provided by S. Jacobson (NINDS, Bethesda, MD). Virus stocks were produced infecting the human T lymphoblast cell line SupT1 (CRL-1942™, American Type Culture Collection, Manassas, VA) for 7 days as described below. At this time, cultures were collected, spun at 756 x g for 7 min, and supernatant kept in aliquots frozen at −80 °C until use. For each virus stock, the amount of protected HHV-6B DNA per mL of supernatant was measured by qPCR as described below; protected DNA is not sensitive to digestion by treatment with DNase-I prior to purification. The values obtained (copies DNA/mL) were used as an indicator of the amount of virus in each stock, and all infections were done considering this parameter when the virus stock dose was determined. Virus was purified from culture supernatant by sequential low-speed centrifugation, followed by ultracentrifugation to obtain a concentrated extracellular virus [22]. The concentrated extracellular virus was loaded onto an Optiprep® (Sigma-Aldrich Inc., St. Louis, MO) step gradient (10-40%) and centrifuged at 160,000 x g for 90 min. Fractions were collected from the bottom and the purified virus was recovered from the fraction containing the peak of HHV-6B strain Z29 specific DNA.

### Infection assays

Cell lines SupT1 and SupT1.CIITA [23], as well as MOLT-3 (CRL-1552™) and Jurkat E6 (TIB-152™) (both American Type Culture Collection, Manassas, VA) were infected with HHV-6B strain Z29. Cells were collected during the exponential phase of growth and incubated with the HHV-6B virus stock. For dose-response experiments, serial dilutions of the virus stocks were used for infection; for time-course experiments, a single dose of 300 copies of virus DNA per cell was used for infection; for virus inactivation experiments, live or inactivated virus, obtained by incubating the virus stock at 56 °C in a water bath for 1 h or under UV light for 1 h, were used. For infection, cells were incubated with the live or inactivated virus stock for 3 h at 37 °C. Cells were then centrifuged and the virus stock aspirated, followed by 2 washes with phosphate saline buffer pH 7.4 (PBS). Finally, cells were resuspended in RPMI 1640 medium supplemented with L-glutamine (2 mM), penicillin (100 U/mL), streptomycin (100 μg/mL) and 5% fetal bovine serum (FBS) at a density of 0.5 × 10^6^ cells/mL and transferred to plates or flasks. At this time, baseline samples (*t* = 0 dpi) were collected. The remaining samples were maintained at 37 °C/5% CO_2_ for the time of infection. At each time point, cell cultures were inspected under the optical inverted microscope (NIKON Eclipse TE2000U equipped with a digital CCD camera) and a sample of the cell suspension was collected for analysis in the imaging-based cell counter. The remaining cell suspension was spun at 756 x g for 7 min, an aliquot of the supernatant was stored at −80 °C for virus DNA isolation and quantitation, and cells were washed with PBS and used for other assays (microscopy, flow cytometry, RNA isolation). HSB-2 cells (CCL-120.1™, American Type Culture Collection, Manassas, VA) were infected by mixing fresh non-infected cells with a cell pellet of HSB-2 cells infected with HHV-6A strain GS at a ratio 5:1, and cultured in Iscove’s Modified Dulbecco’s medium supplemented with L-glutamine (2 mM), penicillin (100 U/mL), streptomycin (100 μg/mL) and 5% fetal bovine serum (FBS) at a density of 0.5 × 10^6^ cells/mL; cultures were inspected every 3 – 4 days and a sample of the cell suspension was analyzed in the cell counter each time.

### Size measurements

Cells suspensions were carefully resuspended by pipetting up and down using a serological pipette, and analyzed using an imaging-based cell counter after mixing 1:1 with 0.2% trypan blue. The Cellometer Bright Field Cell Counter Auto T4 (Nexcelom Biosciences LLC., Lawrence, MA), which uses bright field imaging and pattern-recognition software to identify and count individual cells in 8 different fields of a slide in 20 μl samples was used to collect the data [24]. For SupT1, large size cells parameters (set to measure cell sizes between 8 and 50 μm) were used; same parameters were used for MOLT-3 but adjustments were introduced for measuring SupT1.CIITA (extending the range to 100 μm), Jurkat E6 and HSB-2 (adjusted to include smaller cells). These parameters usually guaranteed all cells observed in the slide were counted. Inspection of the fields before counting was routinely performed, to ensure absence of cell clumps (the software also has a de-clumping function). Raw data obtained consisted in count and size of each live and dead cell; in addition, cell size distribution, cell density, and viability (assessed by trypan blue exclusion assay) were reported by the software.

### DNA extraction and qPCR

DNA was extracted from frozen supernatants, using the QIAmp Minielute Virus Spin kit (QIAGEN Inc., Valencia, CA), following manufacturer’s instructions. Prior to extraction, digestion of un-protected DNA was performed incubating the sample with DNase-I (Roche Diagnostics Corporation, Indianapolis, IN) for 30 min at 37 °C, followed by addition of EDTA (final 15 mM) and incubation at 70 °C for 10 min [25]. This step ensures the quantitation of encapsidated virus only. Real-time qPCR was used to quantify specific HHV-6B strain Z29 DNA, using forward primer 5′-GAG ACC GGG TCT GGA CAA CA-3′; reverse primer 5′-GAG TTG CTG AGT TGG TAA AGG-3′; and probe 6-FAM-CTC CAA GTG TAC CGA AAC GCT TCC TGG–TAMRA (Applied Biosystems, Thermo Fisher Scientific, Inc.); this set of primers and probe amplify a region in the U90 gene. A calibration curve (< 1 - 10^7^ copies DNA per reaction) was obtained using serial dilutions of a plasmid containing the gene amplified by the primer set. Reactions were carried on CFX96 Real Time System (Bio-Rad Laboratories, Inc.). Copies of viral DNA per well were calculated using the measured Ct values and the calibration curve. Amplified product was not observed for dilutions of plasmid containing ≤ 1 copy per well, and we considered this to be our lower limit of detection. An average of 8 ± 2 copies per well were reliably detected with 100% detection rate. Copies of viral DNA amount per mL of supernatant were calculated using the per well values and the appropriate dilution factors, yielding a detection limit of 6 × 10^3^ copies/mL, Ct > 41). In test experiments we found that two washes were sufficient to remove > 90% of the viral inoculum from infected cultures, with an additional wash not further reducing residual virus. Thus for high dose infections, viral DNA in supernatant could be detected early after infection even in the absence of viral replication. This level was essentially constant for measurements at day 0 and day 1, with significant increases (> 30-fold) observed for day 2 and beyond.

### Flow cytometry

Monoclonal antibody anti-HHV-6 gp116/64/54 (Clone 6A5G3), recognizing a late antigen (glycoprotein B, gB), was obtained from the HHV-6 Foundation Reagent Repository. Non-infected and infected SupT1 were stained at 4 and 7 days post-infection (dpi) with anti-gB (dilution 1:100) after fixation and permeabilization using the CytoFix/CytoPerm kit (BD Biosciences, San Jose, CA). Goat-anti-mouse-IgG (Fab)-FITC (KPL, Inc., Gaithersburg, MD) or goat-anti-mouse Ig-BV421 (BD Biosciences, San Jose, CA) were used as secondary antibodies. Unstained cells and cells stained with isotype control antibody (IgG2b; BD Biosciences, San Jose, CA) were used as controls. Data were acquired using a BD LRSII flow cytometer equipped with BD FACSDiva software (BD Biosciences, San Jose, CA) and analyzed using FlowJo v. 10.1 (FlowJo, LLC, Ashland, OR). Dead cells and cell debris were gated out and FITC or BV421 fluorescence was determined in live, single cells. Gates were set considering live cells and background fluorescence from non-infected cells stained with the virus-specific antibodies, from infected cells stained with isotype control, and from auto-fluorescence of infected cells.

### RNA extraction and RT-PCR

SupT1 non-infected or infected with live or inactivated HHV-6B strain Z29 were used to verify transcription of viral genes at 4 and 7 dpi. At the various time points cells were collected by centrifugation, washed with PBS and total RNA was extracted using the RNeasy kit (QIAGEN Inc., Valencia, CA), following manufacturer’s instructions. The procedure included a DNase-I digestion step, to digest any free virus DNA present in the preparation. First strand DNA synthesis was performed using RevertAid First Strand cDNA Synthesis kit (Thermo Scientific, Inc.), starting from ~ 1 μg total RNA. RT-PCR was performed using Dream Taq Green PCR Master mix (Thermo Scientific, Inc.) and primers for two virus transcripts (U86 and U12) and beta-actin as reference gene (all primers sequences from De Bolle et al. [26]).

### Electron microscopy

Electron microscopy analysis was performed by the Core Electron Microscopy Facility (University of Massachusetts Medical School, Worcester, MA). Infected and non-infected cells (prepared following directions from the facility) were analyzed by transmission electron microscopy (TEM) and scanning electron microscopy (SEM). Virus purified from supernatant of infected cells was analyzed by negative staining.

### Statistical analysis

Statistical analyses were performed using PRISM v7 (GraphPad Software, Inc.). For comparison among groups t-test or Mann-Whitney test (2 groups) or ANOVA (3 or more groups) were used. Pearson product-moment correlation coefficient was used to evaluate linear correlation between variables; linear regression was done using the least squares fit. Receiver operating characteristic (ROC) analysis was used to evaluate the performance of the methods and select cutoff values. Cutoff values were selected maximizing the product of specificity and sensitivity [27].

## Results

### Characterization of the cytopathic effect induced by HHV-6B infection in SupT1 cells

**Fig. 1:**
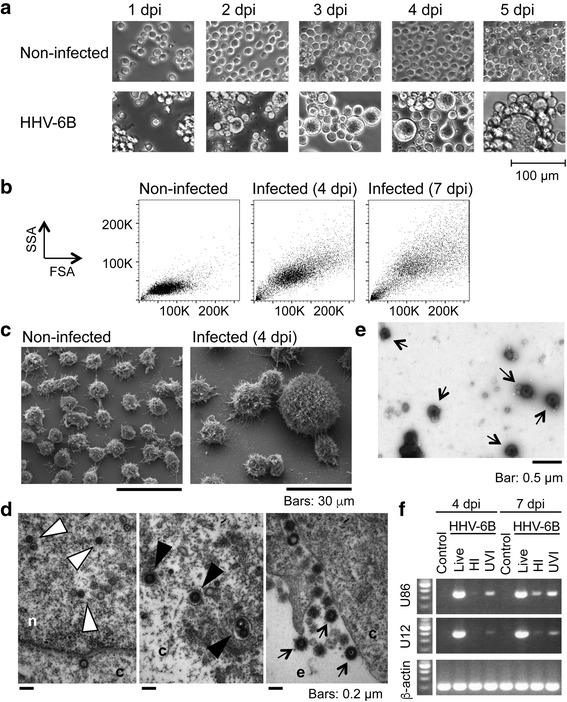
Cytopathic effect in HHV-6B-infected SupT1. **a**. Phase-contrast microscopy images (20×) of SupT1 cells suspension non-infected and infected at different days post-infection (dpi); bar = 100 μm. **b**. Flow cytometry scatter plots (FSA vs SSA) of non-infected and infected SupT1 cells at 4 and 7 dpi. ***C****. SEM* images (2,000×) of non-infected and infected SupT1 at 4 dpi. Bar: 30 μm. **d**. TEM images (43,000×) of infected SupT1, showing different stages of virus replication: nucleocapsids in the nucleus (*white arrowheads*), tegumented virus acquiring envelope in the cytoplasm (*black arrowheads*), and virions in the extracellular space (*arrows*); bars = 0.2 μm. **e**. Negative staining (20,500×) of virus purified from supernatant of infected cells; *arrows* show virions; bar = 0.5 μm. **f**. Viral gene expression in SupT1 non-infected or infected with live or inactivated virus (HI = heat-inactivated; UVI = UV-inactivated) at 4 and 7 dpi. Agarose gel shows PCR products for U86 (*top*), U12 (*middle*) and beta-actin (*bottom*) transcripts for each condition

In order to verify that the infection with HHV-6B virus induced the characteristic cytopathic effect in SupT1 cells, i.e. the occurrence of enlarged cells, cells were infected with HHV-6B strain Z29 at a ratio of 300 copies DNA per cell and the cultures were monitored daily for up to 7 days. Figure 1a shows phase-contrast optical microscopy images of non-infected SupT1 and SupT1 infected with HHV-6B after 1 to 5 days post-infection (dpi). Enlarged cells can be already observed at 2 dpi, and the number and size of these cells increased with time. Cytopathic effect can also be appreciated by flow cytometry, in forward (FSA) versus side (SSA) scatter plots. In the example presented in Fig. 1b, increase in both size (FSA) and granularity (SSA) of infected cells at 4 and 7 dpi is evidenced by a shift of the cells’ distribution towards higher values. The striking differences in size can be fully appreciated in Scanning Electron Microscopy (SEM) images, where the homogeneous population of small non-infected cells gives rise to a population containing bigger cells of heterogeneous size, including some giant cells (Fig. 1c).

To confirm that the presence of enlarged SupT1 cells was indeed a result of a productive infection and not a result of the “fusion from without” phenomenon [28], samples were analyzed for active replication and production of virions. Ultra-thin slices of infected cells analyzed by Transmission Electron Microscopy (TEM) (Fig. 1d) showed evidence of the production of virions. Capsids were identified in the nucleus (white arrowhead), and viral particles at different stages of maturation (black arrowhead) were observed in the cytoplasm as well as in the extracellular space (black arrows). Extracellular virus was present in the supernatant of infected cells, as evidenced by negative staining of virus purified from the cell culture supernatant (Fig. 1e, black arrows); the virus in these supernatants was infectious, as indicated by their ability to infect fresh non-infected cells. Moreover, virus genome replication was verified by detection of increased levels of virus DNA produced (~ 1.5 × 10^9^ copies/mL in cultures infected with live virus vs. < 5 × 10^5^ copies/mL in cultures infected with inactivated virus). Further, expression of two viral transcripts (from genes U86 and U12) was evaluated, confirming that cells infected with live virus expressed high levels of these transcripts (Fig. 1f), while non-infected cells or cells infected with inactivated virus showed no or low-levels of expression of the transcripts. All these results confirm that SupT1 cells were productively infected by HHV-6B.

### Assessment of HHV-6B infection by measuring the size of cells

Given the occurrence of enlarged cells in cultures of SupT1 infected with HHV-6B, we investigated whether the measurement of the size of the cells and the number of enlarged cells could be used as an indicator of the level of infection of the culture. One method to measure the size of cells is using an imaging-based automated cell counter. In this work, we use a Cellometer Auto T4 instrument for bright field image capture and analysis (Nexcelom Biosciences LLC.). This instrument essentially is a low-resolution imaging microscope that uses bright field imaging and pattern-recognition software to identify and count individual cells [24].

**Fig. 2:**
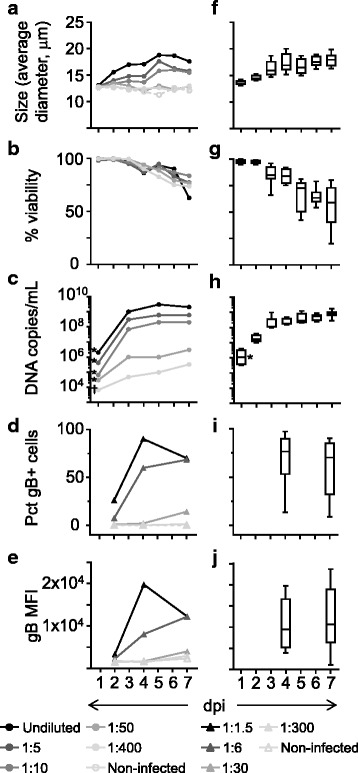
Characterization of HHV-6B-infection by different methods. Time-course of cells infected with different doses of a virus stock, measured at different times: **a**. Average diameter of cells (μm); **b**. Cell viability (%); **c**. Virus DNA in supernatant (copies/mL); **d**. Percentage of glycoprotein B positive cells (Pct gB+ cells); **e**. gB median fluorescence intensity (gB MFI). Summary of time-course experiments of SupT1 infected with different virus stocks at a single dose of virus (300 copies per cell): **f**. Average size; **g**. Cell viability; **h**. Virus DNA in supernatant; **i**. Pct gB+ cells; **j**. gB MFI. Box plots show median, quartiles, and standard deviation of 7-10 independent infections. * Day 1 values in panels **c** and **h** reflect residual viral DNA present despite extensive washing. ^†^ Limit of detection

In order to assess whether the use of the imaging-based automated cell counter was suitable to obtain an objective and accurate measurement of the size of cells infected by HHV-6B, SupT1 cells infected with different doses of the virus and followed for up to 7 days were analyzed. The average size of cells in culture and the viability were measured daily. For comparison and validation, HHV-6B infection was also assessed by other methods. The amount of protected virus DNA in supernatant was quantified by qPCR and the percentage of cells expressing a viral glycoprotein (gB) was measured by flow cytometry. Results are shown in Figs. 2a-e. The average size of cells in the infected cultures progressively increased after infection, and the increases in average size were more marked when higher amounts of virus were used (Fig. 2a). For low doses of virus, only very slight changes in average size of the cultures were observed, which were similar to the slight changes observed in non-infected cells. In terms of viability of the cultures, a progressive declined after 3 dpi was observed (Fig. 2b). Furthermore, quantification of virus DNA in supernatants showed a progressive increase with time of virus DNA accumulated in supernatants for all doses of virus tested. Higher doses resulted in larger increments of virus DNA in supernatant compared to the residual amount at day 0 (> 100-fold at 3 dpi), while lower doses resulted in smaller increments (Fig. 2c). In addition, both the percentage of infected cells positive for gB expression (Fig. 2d), and the median fluorescence intensity (MFI, Fig. 2e), were higher for cultures infected with higher doses of virus. Expression of gB in cells increased with time at all doses tested, with the exception of the highest dose at the last time point for which a decrease was observed. In general, doses of virus that induced marked increases in the amount of DNA accumulated in supernatant and the expression of gB in cells also induced measurable increases of the average size of the cells in infected cultures.

Figs. 2f-j summarizes the results of infections using different virus stocks at a single dose of virus (300 copies of DNA per cell, *n* = 7-10 independent infections). Results confirm the trend observed before: the average size of cultures progressively increased after infection (Fig. 2f). Increases were statistically significant at and after 3 dpi compared to the initial average size. By 3 dpi the average size increased about ~ 30%; by 7 dpi an additional 15% increase was observed. From 3 dpi, the viability of the cells progressively declined, and the decrease was significant at and after 5 dpi (Fig. 2g). The amount of virus DNA released into the supernatant increased more than 100 times from the residual amount of the virus measured at day 0 during the first 3 days of infection; then, it reached a plateau, with an additional increase of ~ 2-3 times by 7 dpi (Fig. 2h). The expression of gB was increased at both 4 and 7 dpi, however with a large variability in the measurements (Figs. 2i-j). The trends were consistent for all virus stocks tested, with the differences observed possibly related to the actual infectivity of the different stocks (stocks were used based on copies of virus DNA/mL, which does not always correlate with infectivity). Finally, the effect of virus inactivation prior to infection was tested. When infection of SupT1 with live virus was compared to infection with heat-inactivated or UV-inactivated virus, a substantial increase in the average size of the cells was observed only for the live virus (see Additional file 1), indicating that a productive infection is indeed necessary for the occurrence of the enlarged cells in this system. Overall, these results show that an imaging-based automated cell counter is able to reveal differences induced by infection at different doses and times. Measurements of the average size of cells in infected cultures follow a similar trend as other methods used to measure the infection, suggesting that the size measurements can indeed be useful to monitor and characterize HHV-6B infections.

### Size measurements in non-infected and HHV-6B-infected SupT1 cells

**Fig. 3:**
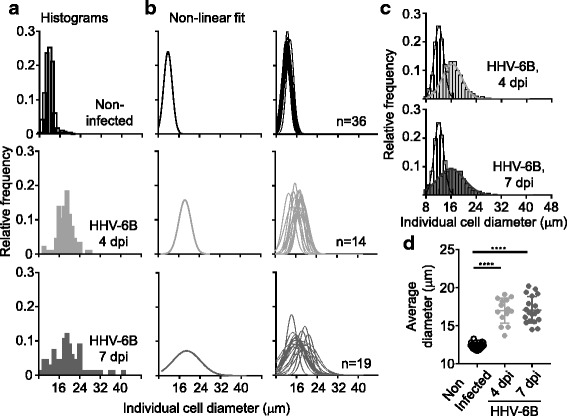
Assessment of HHV-6B infections using size measurements. **a**. Example of raw data obtained from a single sample of non-infected or infected cells at 4 and 7 dpi, graphed as a histogram of cell sizes. **b**. Non-linear fit of the data at 7 dpi for a single replicate (*left*), and for data from 36 non-infected, 14 infected at 4 dpi and 19 infected samples (*right*). **c**. Histograms and non-linear fit of size data from non-infected (open bars) and infected (*light gray* (4 dpi) and *dark gray* (7 dpi)) cells; multiple replicates within a population were combined. **d**. Average size of cells for different replicates within a population (non-infected, 4 and 7 dpi, same color scheme described before); statistical significance levels (ANOVA) are presented

In order to study the ability of the size measurement-based method to distinguish non-infected and infected cells and cultures, analysis of three populations of cells was performed: infected cells at 4 dpi (representing the earliest time point where the different indicators of infections reached a maximum); infected cells at 7 dpi (which is the standard time to collect supernatant during the production of virus); and non-infected SupT1, usually collected at 4 days after a new culture was established. Data from multiple replicates (independent infections using different virus stocks) were collected over months (non-infected *n* = 36; 4 dpi *n* = 14; 7 dpi *n* = 19). The raw data collected using the imaging-based cell counter was used to generate size histograms for each sample (Fig. 3a), and non-linear fits of the raw data to Gaussian curves were calculated Fig. 3b). These results showed that non-infected cells comprised a relatively narrow population in size, that for infected cells a broad ranges of sizes was observed, and that the overall size populations were shifted dramatically upon infection.

In order to compare the three populations, two approaches were used. In one approach, raw data (size of each cell) from the different replicates within a population were combined and the histograms and fits for the three populations were obtained (Fig. 3c). When the populations were compared, a shift toward larger sizes in infected cells was observed, along with an overlap between the non-infected and infected populations. In the other approach, the processed data (average size of cells) was used. For instance, for each replicate instead of using the size of individual cells, the average size of all cells counted in that sample was calculated; then, values from different replicates within each population were used for the comparisons (Fig. 3d). The use of the parameter “average size of the cells” resulted in a clear separation (less overlap) between non-infected and the two infected cell populations. Statistically significant differences were found between non-infected and the two infected populations (although not between 4 and 7 dpi populations).

We explored the feasibility of using average size measurements to evaluate HHV-6 infection in other systems. We tested the human T lymphoblast cell lines SupT1.CIITA, MOLT-3, and Jurkat E6 for infection with HHV-6B strain Z29, and the human T- lymphoblast cell line HSB-2 for infection with HHV-6A strain GS. For all combinations of cell lines and virus strains tested, a measurable shift in the average size of infected cells compared to non-infected cells was observed (Additional file 2). The susceptibility of different cell lines to cytopathic effects after infection was variable, depending on the combination of cell line and virus as well as doses of virus and time post-infection (not shown). Also, the average size of the non-infected cultures was slightly different for the different cell lines. Interestingly, the cell line SupT1.CIITA was prone to generate a high proportion of cells larger than the observed in SupT1 (>100 μm), which also appeared at shorter times (2 dpi); the analysis of samples containing a high proportion of these cells was more complicated and not accurate, as these cells were hard to sample homogeneously. Overall, it appears that this method should be applicable to other systems, but optimization for each case likely would be necessary.

### Performance of size measurements in differentiating non-infected from infected SupT1 cells and cultures

**Fig. 4:**
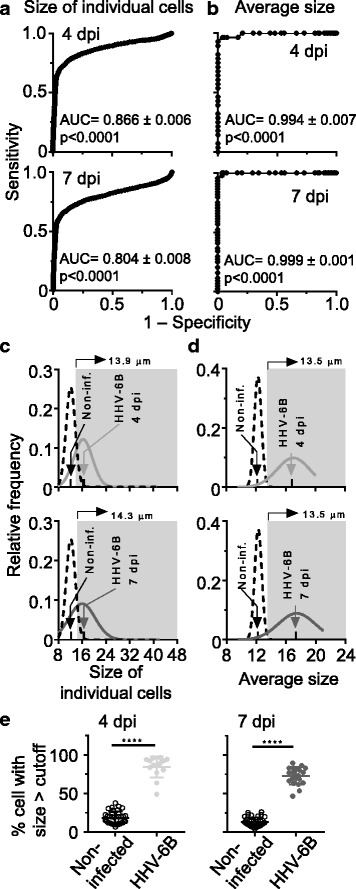
Performance of size measurements as a method to differentiate non-infected from infected cells and/or cultures. ROC curves obtained for size of individual cells (**a**) and for average size of cells (**b**), using a non-infected culture as control and infected cultures at 4 or 7 dpi as experimental conditions. The AUC and significance level are shown in each case. Non-linear fit for each pair of data analyzed showing the cutoff selected when the analysis was done using size of individual cells (**c**) or average size of cells (**d**) at 4 or 7 dpi. The shaded area comprises the range of sizes for infected cells or cultures at the selected cutoffs. **e**. Estimation of the percentage of infected cells derived from the analysis using size of individual cells. Statistical significance level determined by Mann-Whitney

We used ROC (receiver-operating characteristic) analysis [29] to evaluate the performance of size measurements as a method to differentiate non-infected and infected cells and/or cultures. ROC curves show the tradeoff between sensitivity and specificity. Sensitivity is the ability to detect a positive response; an assay with high sensitivity would provide few false negatives. Specificity is the ability to exclude negative responses; an assay with high sensitivity would identify few false positives. An ideal assay provides high specificity and high sensitivity, but development of a practical assay involves tradeoffs between these. ROC curves were calculated for both of the measurements, size of individual cells and average size of cells in culture, and are shown in Fig. 4a and b for 4 and 7 dpi respectively. We used measurements from non-infected cells as negative controls and data from infected cells and/or cultures as experimental conditions. An ROC curve for an ideal assay is a vertical line on the y-axis (at specificity = 1.0) with a horizontal line at sensitivity = 1.0. A ROC curve for an assay that is not better than random prediction is a diagonal line. It is apparent that both measurement approaches have great power in differentiating non-infected and infected cells or cultures, in particular when the average size (Fig. 4b) was used.

The area under the ROC curve (AUC) represents the probability of correctly identifying a positive sample, specifically the probability that a randomly selected true positive will have a higher predicted value than a randomly selected true negative. The AUC for an ideal assay is 1.0 and for a random assay is 0.5. AUC values for both measurements, individual size and average size, are also shown in Fig. 4a and b. Measurement of the average size performance approximates the ideal. For the individual size measurements, there is a trade-off between specificity and sensitivity, because of the overlap in the size-distribution profiles (Fig. 4c). A cutoff that includes all infected cells (gray histogram) will necessarily include some non-infected cells (dashed histogram). Size distribution profiles for measurements of the average size (Fig. 4d) overlap to a much lesser degree.

ROC analysis can be used to identify an optimum cutoff value. In order to optimize the proportion of false positives and false negatives, we identified a suitable cutoff value by maximizing the product of sensitivity and specificity [27]. The cutoff values calculated define regions in the size distribution plots (shaded area in Fig. 4c and d), which were expected to contain infected cells or cultures with high confidence. With this cutoff, the average size was associated with a low overlap between the populations, which correspond to lower percentages of misidentifications. On the other hand, size of individual cells has higher overlap and higher percentage of misidentifications. The calculated cutoff values, level of sensitivity and specificity, and the rates of false positives and negatives are shown in Table 1. For simplicity, consensus values for both approaches were also selected; the trade-off associated to both sensitivity and specificity using the consensus values also is reported in Table 1.

**Table 1:** Sensitivity and specificity for selected cutoff values

Size measurement approach	Size of individual cells		Average size	
Infection day (dpi)	4	7	4	7
Cutoff value (μm)^a^	13.9	14.3 (13.9)	14.2 (13.5)	13.5
Sensitivity^b^	0.77	0.66 (0.69)	0.93 (0.96)	1.00
False negatives^c^	23%	34% (31%)	7% (4%)	0%
Specificity^b^	0.87	0.91 (0.87)	1.00 (0.96)	0.96
False positives^c^	13%	9% (13%)	0% (4%)	4%

^a^Cutoff value for maximized product of sensitivity and specificity for each size measurement approach and infection day (dpi); in parenthesis, a consensus cutoff value for each size measurement approach is shown. ^b^Sensitivity and specificity at each cutoff value; in parenthesis, sensitivity and specificity associated with the consensus cutoff values. ^c^Percentage of false negatives and false positives at each cutoff values; in parenthesis, values for consensus cutoff values

We tested this approach in evaluating a large set of experimental infections used for virus production with different virus stocks performed on different days. The fraction of enlarged cells was defined using the cutoff values described above. Fig. 4e shows the percentage of enlarged cells calculated for non-infected and infected samples. There is a clear difference in the percentage of cells above the cutoff between non-infected and infected samples; Mann-Whitney analysis shows the differences are statistically significant under these conditions. This result suggests that this approach could also be used to differentiate non-infected from infected cultures. A ROC analysis of this experiment is shown in Additional file 3.

### Correlation of size-based method with other methods

**Fig. 5:**
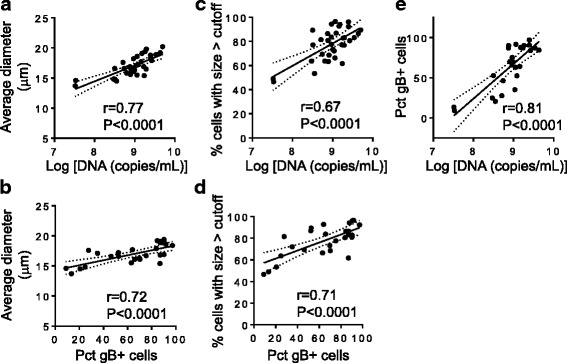
Validation of size measurements. Correlation analysis performed between average size and virus DNA in supernatant (**a**) or average size and percentage of gB positive cells (Pct gB+ cells) (**b**); correlation between percentage of cells above the cutoff size and virus DNA in supernatant (**c**) or percentage of cells above the cutoff size and Pct gB+ cells (**d**); and correlation between Pct gB+ cells and DNA in supernatant (**e**). Pearson’s correlation coefficients and statistical significance levels are shown

In order to validate the method based on size measurements as suitable way to estimate infection, correlation analysis between size-based method and two other methods (levels of virus DNA in supernatant and percentage of cells expressing gB) was performed. Correlation of average size of cells in culture vs. copies of virus DNA/mL and vs. percentage of cells expressing gB are shown in Fig. 5a and b, respectively. Correlation of percentage of cells with size above the cutoff vs. copies of virus DNA/mL and vs. percentage of cells expressing gB are shown in Fig. 5c and d, respectively. Correlation between copies of virus DNA/mL and percentage of cells expressing gB is shown in Fig. 5e. Significant positive moderate correlations were observed in all cases. Correlations using average size of cells in culture were similar or better than correlations using percentage of cells with size above the cutoff. Altogether these results indicate that all three methods depict a similar trend when used to estimate the level of infection.

To explore the feasibility of using the percentage of infected cells calculated by the size analysis to predict actual percentage of cells expressing viral antigens, linear regression analysis was performed. The coefficient of determination (r^2^) was 0.51, which indicates that about 51% of the data on antigen expression can be explained by the data on percentage of cells with size above the cutoff values. The standard error of the estimate (Sy.x) was 20.0%; the error in predicting the percentage of cells expressing viral antigens was slightly lower than the standard deviation for the measurements of percentage of cells positive for the antigen (64.1 ± 27.5); these results suggest a moderate accuracy of the predictions.

### Relative sensitivity range of size-based method

**Fig. 6:**
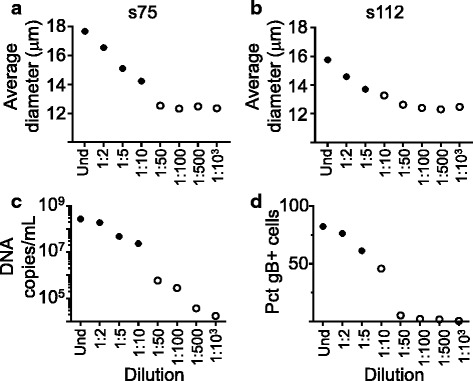
Relative sensitivity range of size, qPCR and flow cytometry measurements. **a**-**b**. Dilution curve for two virus stocks, using the average size as a method to measure level of infection (A = s75, B = s112); broken line indicates cutoff value for the size method (13.5 μm). **c**. Dilution curve of s75 using qPCR (copies of DNA/mL in supernatant) as measurement of infection. **d**. Dilution curve of s112 using percentage of gB positive cells (Pct gB+ cells) as measurement of infection; broken line indicates the cutoff value (4.7%). In all graphs, open symbols indicate dilutions that were categorized as negative by the size-based method

To test the range of sensitivity of the method, cultures of cells infected with serial dilutions of virus stocks were monitored at 4 dpi, measuring the average size of the cells, DNA copies/mL by qPCR, and percentage of cells expressing gB by flow cytometry. Infecting cells with increasing dilutions of the virus stock resulted in reduction in the average size of the cells relative to that observed for the undiluted virus stock (Fig. 6). For the two viral stocks tested in the experiment shown (s75 in Fig. 6a and s112 in Fig. 6b) the average cell diameter did not decrease for dilutions below 1:50. The average size of cells for these dilutions was below the cutoff value calculated at 4 dpi (13.5 μm, broken line in Fig. 6a and b), which suggests that these samples can be considered as negative by the size-based method. Figure 6c shows amount of virus DNA in supernatant for the s75 infection and Fig. 6d shows percentage of gB in cells for the s112 infection. As observed with the average size, increasing dilution resulted in lower copies of DNA/mL in supernatant and lower percentages of cells expressing gB. However, for dilutions categorized as negative by the size-based method (open circles in Fig. 6), changes in copies of DNA/mL and to a lesser extent expression of gB could be clearly detected. This indicates a lower sensitivity in the size-based method as compared to the flow cytometry and qPCR assays.

### Measuring infectivity using size-based method

**Fig. 7:**
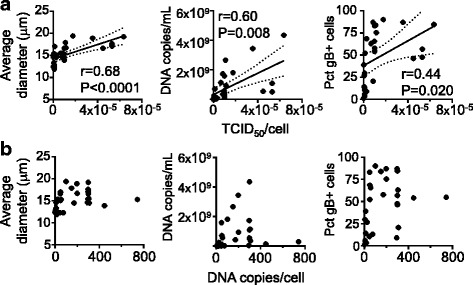
Normalization of the dose of virus in different virus stocks. Correlation of dose of virus calculated using TCID_50_/cell (**a**) or copies of DNA per cell (**b**) and parameters used to estimate infection of the resulting culture (average size, DNA in supernatant (copies/mL) and percentage of gB positive cells (Pct gB+ cells). Pearson’s correlation coefficients and statistical significance levels are shown

We evaluated whether size-based tissue culture infectious dose (TCID_50_) measurements provide an effective tool for determining the relative infectivity of viral stocks. Infectivity of virus stocks usually is assessed by end-point dilution assay, which allows the calculation of the TCID_50_ and the associated titer, using the method described by Reed and Muench [30]. The read-outs generally used for determination of relative infectivity are visual inspection of cultures for identification of enlarged cells; IFA or flow cytometry to detect expression of viral gene products on infected cells; or recently, Gustafsson et al. described a method that uses qPCR measurements as read-out of infection for TCID_50_ assays [31]. In order to evaluate the size-based method as a read out for TCID_50_, an end-point dilution assay was set up. We infected SupT1 cells with serial dilutions of viral stocks (4 wells per dilution) measuring the average size of cells in each well at 4 dpi. Infected cultures exhibiting average cell size higher than the cutoff value (13.5 μm) were considered positive infections. The method was used to estimate the infectious potential of 12 virus stocks, with values reported as TCID_50_/mL; the same stocks were evaluated for copies of viral DNA/mL using qPCR (Table 2). Next, we used the 12 different HHV-6B stocks to infect SupT1 cells at various doses of virus, monitoring infection using average size as well as qPCR and flow cytometry assays (Fig. 7a). We observed good correlation between the level of infection measured by any of the methods and the dose of the various stocks calculated using the size-based TCID_50_/mL. We compared these values to those determined using DNA copies/mL instead of TCID_50_/mL as a measure of the dose virus in the various infections. In contrast to the results using TCID_50_ per cell, no significant correlation was observed between measures of infection and DNA copies per cell used to infect the cultures (Fig. 7b). This result suggests that level of infection of a culture measured as copies of DNA/mL in supernatant does not necessarily reflect the infectious potential of that supernatant (i.e. amount of infectious virus). Overall, TCID_50_/mL calculated using the size-based method is an easily obtained and reliable tool to normalize virus stocks for infection cultures.

**Table 2:** TCID_50_ of HHV-6B Z29 stocks by end-point dilution assay, using size measurements as read-out

Stock Id	TCID_50_/mL by average size^a^	Virus DNA^b^ (copies/mL)
s75	45	3.0E + 08
s77	3	3.5E + 08
s78n	34	1.2E + 09
s81	447	2.5E + 09
s83	141	4.9E + 09
s84	447	2.5E + 09
s88	141	1.9E + 09
s100	50	1.8E + 09
s102	159	7.5E + 08
s106	45	2.0E + 09
s111	58	4.4E + 09
s112	20	1.1E + 09

^a^TCID_50_/mL calculated using Reed-Muench method [30] and average size of cells as a read-out. ^b^Concentration of virus DNA in the virus stock measured by qPCR

## Discussion

This paper reports the development and evaluation of an assay useful for day-to-day follow-up of HHV-6B infections in-vitro. It was demonstrated that the cytopathic effect observed in HHV-6B infections (occurrence of enlarged cells) can be measured using an imaging-based automated cell counter, which provides systematic and objective measurements of the size of cells in the cultures. As the occurrence of enlarged cells was associated to productive infections in this particular system, this method provides an objective, fair, and fast way to monitor infections. The cell counter measures the diameters of cells identified in 8 different fields of a slide, providing the count of live and dead cells and their sizes, as well as processed data like cell density, average cell size and cell viability. The dynamic range for the average size of cultures was relatively narrow, with the settings of the counter selected for counting individual cells of up to 50 μm; conveniently, this parameter can be easily adjusted which broaden the applicability to other systems.

Results obtained from testing the method in the T lymphoblast cell line SupT1, susceptible to infection by HHV-6B strain Z29, indicate that it can indeed be used to differentiate non-infected and infected cells and cultures. However, analysis of the size of individual cells showed some overlap between distributions of non-infected and infected populations. It is possible that, as a result of a non-synchronic infection of cells, the fraction of cells that were not infected, or which were not yet displaying cytopathic effect, might account for some degree of this overlap; this implies at least two populations coexisting in the same culture. In addition, non-infected cells undergo slight variations in size, likely related to the cell-cycle stage, also contributing to the overlap, although to a lesser degree. The overlap is greatly reduced when the average size of cells is used for the analysis rather than the distribution individual cell sizes. At the times studied (4 and 7 dpi) enlarged cells are likely predominant, driving the average to higher values and reducing the overlap. Indeed, there were minimal doses of virus and minimal times post-infection before which the infected cultures could not be assessed by the method. It is possible that under conditions of low virus input, non-infected and relatively fast-dividing cells outnumbered the few infected cells initially generated, resulting few enlarged cells and neglectable changes in overall average size of the cells. Low amounts of virus produced under these conditions would not generate enough infected cells to induce a measurable shift in the population’ size distribution. Likewise, at earlier time-points, the increase in average size and/or the number of enlarged cells might not be big enough to generate a significant shift in the population’ size distribution. As infections progress, the proportion of infected cells and the actual size of these cells would have increased enough to significantly shift the average size of the cells.

A ROC statistical analysis was used to define a practical cutoff value for deciding whether there was infection or not. The performance of the method was better when the average size of cells rather than individual cells’ size distributions were used. The selection of the cutoff value was done by maximizing the product specificity and sensitivity and considering how much loss of specificity (how many false positives) or loss in sensitivity (how many false negatives) can be tolerated. Note that these depend on the particular aim of an experiment. The consensus cutoff values selected for the analysis involving average size of cells were associated with 4% or less false negatives and false positives, which was considered appropriate. In the case of monitoring production of virus stocks for example, not reaching 100% sensitivity might be acceptable, as infected cultures that cannot be clearly differentiated from the non-infected cultures are likely to represent low-level infections, and resulting virus stocks likely to contain low amount of virus. In the case of measuring the infectivity of a virus stock, both the highest specificity and sensitivity are desirable, because wrong assignment of positive or negative wells would affect the determination. The performance of the method when individual size of cells was used was not as good as for average size of the population, which might be a reflection of the nature of system (inherent size variation in non-infected cells and non-synchronic infections) resulting in a mix of cells at different states. The cutoff values selected when using individual size of cells were associated with higher percentages of false negatives (23-34%), which indicates that cells below the cutoff value might be wrongly assigned as not infected. This is consistent with the results of the linear regression analysis, in which only 51% the data on antigen expression in cells was explained by the percentage of cells above the cutoff.

The size-based method described here was particularly useful when characterizing medium and high level infections; unfortunately, low-level infections did not generate enough enlarged cells such that the shift in the size distribution could not be differentiated by this method. For instance, in procedures such as generation of infectious virus stocks or production of infected cells (for preparation of total cell lysates for protein analyses, gene expression assays, etc.), the size-based method can be used to follow the infections. In these cases, inability of detect low-level infections does not reduce the practicality of the method, given that low-level infections do not result in good stocks able to further propagate the virus nor in highly infected cell preparations, where any change in gene or protein expression in infected cells would not be masked by the expression in the predominant non-infected cells.

The analyses and cutoff values described in this work were obtained using the system of SupT1 cells and HHV-6B strain Z29 virus. Other cell lines and virus, such as SupT1.CIITA [23], MOLT-3 and Jurkat E6 infected with HHV-6B strain Z29 and HSB-2 cells infected with HHV-6A strain GS, also showed measurable occurrence of enlarged cells, which should make them suitable for analysis by this method. However, different combinations of cell types and virus strains have specific characteristics. Basal (non-infected) size distributions were different for the different cell lines, as were infection susceptibilities. Thus, each combination of cell and virus need to be individually standardized. In setting up the assays, some factors discussed above should be considered to successfully differentiate non-infected and infected cultures, in particular the time after infection when data is collected, the initial amount of virus used for infection and confirmation of a productive infection. HHV-6 is known to infect many cells type with infection progressing to different endpoints in different cells and with different degrees of viral production [26]. It is possible that certain cell types might be infected by HHV-6B without exhibiting cytopathic effects or size increases upon infection. The method described here would not be appropriate for such situations.

Another interesting observation was the capacity of the size-based method to measure the effect of various methods of virus inactivation. It is known that heat and UV-inactivation have an effect in the infectivity of HHV-6, interfering with processes that require the establishment of a productive infection, as shown by greatly reduced antigen expression and cytopathic effect [32, 33]. However, these inactivation methods do not eliminate the ability of the virus to induce other processes, for example cytokine and chemokine secretion [34, 35], suggesting that stable components of the virion are preserved and can still be taken up by cells and exert effects. Under the conditions employed in our experiments, there was a significant reduction of the amount of infectious virus after inactivation; although not complete, it was enough to result in low-level infections, which did not induce changes in the average size. Bearing this in mind, the size-base method potentially could be used as a first screening tool to test antiviral methods.

In our work, we used a particular microscope-based imaging cell counting system but, in principle, the size-based method would not be restricted to imaging instruments and other counting methods should work as well. Flow-based cell counters, like Coulter counters and flow cytometers, also collect size-based data, and potentially could also be used for this type of analysis. In a flow cytometer for example, the forward scatter data (FSA) could be used for quantitative size-based assessment of infections. A preliminary assessment of this method showed a shift in the population of infected cells, as observed in FSA histograms (Additional file 4). However, we observed that it was important to carefully and consistently select the voltages to ensure that most of the population of infected cells is included in the analysis and to allow comparisons among measurements performed at different days. A second important point to consider in the use of a flow-based cytometer in evaluation of infection-induced size increases is the increased susceptibility of giant cells to fragmentation, which has been noted in a previous study [36]. In that report, researchers used a Coulter counter, and found that, enlarged cells that they could identify by visual inspection using a microscope could not be seen in the size histogram obtained by the Coulter counter, leading them to suggest that the big refractile infected cells had been fragmented during the process [36]. In this regard, a non-flow imaging cell counter such as the one use in our work reported here, has the advantage that the sample manipulation is minimal, favoring the preservation of the bigger cells to a certain degree.

In comparisons of measurements of average size with measurements of the amount of virus DNA in supernatant or the expression of viral antigens in cells, similar trends were observed, suggesting that all methods depict the same phenomenon in a similar way. This corroborates that the average cell size-based method is a valid method to follow HHV-6B infections; it has the advantage of being simple, fast, and providing an objective measurable result, all of which can be important when rapid decision is required.

Of the methods that we evaluated, the qPCR-based method was the most sensitive, allowing detection of 8 ± 2 copies of DNA in a single reaction, with a dynamic range between ~10^4^ to ~10^11^ copies of DNA per mL. This allowed detection of small increases in the amount of virus DNA produced when lower amounts of virus were used to infect the cells and/or when earlier times were analyzed. In fact, qPCR method could detect amounts of virus that did not induce measurable shifts in the average size of the cell cultures, consistent with low-level infections. However, this method can be used only for populations of cells, not for evaluation of infection on a cell-by-cell basis.

The flow cytometry method using fluorescence detection of viral antigens has the advantage of providing the frequency of cells expressing that viral antigen within the whole population, which is an important metric when studying viral infections in-vitro. Infected cells could be identified by flow cytometry when relatively low doses of virus and early time points were analyzed, even after accounting for non-specific binding of antibodies to non-infected cells, and by the increased auto-fluorescence of infected cells. A drawback of this method is that infected cells might be mistakenly excluded from the analysis as a result of several factors including the acquisition voltages and gating strategies excluding the largest cells, uptake of viability dyes by dying infected cells, or increased fragility of larger cells. These factors could be especially important when collecting data at longer time points after infection when the viability of the cells has decreased and when there is a high proportion of larger cells. We seem to have observed this effect in Fig. 2d and e, where infections performed with the highest concentration of virus exhibited reduced gB expression at 7 dpi relative to 4 dpi, whereas the other markers of infection (size, cell death, and viral DNA copies) all increase during this time period. This might be the result of the largest and/or most fragile cells being excluded from the analysis.

Finally, besides monitoring infections, the method was used to determine the relative infectivity of virus stocks. In the end-point assay, the average size method performed well in differentiating among dilutions. However, the fact that low-level infections do not induce significant changes in the average size could lead to underestimation of viral titers, especially compared to more sensitive methods like qPCR and flow cytometry. In spite of this, in terms of infectivity, the size-based TCID_50_ provided better equivalence among virus stocks than copies of viral DNA.

## Conclusions

A method is presented to evaluate HHV-6B infection of cell cultures using individual and average measurements of cell size obtained with an imaging-based automated cell counter. Overall, the reciprocity observed in the trends between average size, level of virus DNA in supernatant, and expression of viral antigens in cells, suggests that measurement of average cell size is a suitable method to follow a productive HHV-6B infection, particularly at higher doses of virus and/or longer times post-infection. The method may prove useful for a rapid initial assessment of infected cultures with the advantage of generating almost immediate results.

## Additional files

Additional file 1: Effect of heat-inactivation and UV-inactivation of HHV-6B in the average size of cells. Control: non-infected SupT1; HHV-6B: live, heat-inactivated, UV-inactivated. Statistical significant differences observed only between control and live virus. (PDF 76 kb)

Additional file 2: A. Phase-contrast microscopy images (20×) of SupT1.CIITA, MOLT-3 and Jurkat E6 cells non-infected or infected with HHV-6B strain Z29 (4 dpi); the right panel shows size histograms of non-infected cells (clear bars) and infected cells (gray bars) for each of the aforementioned cell lines; also shown are the non-linear fits for each sample (dashed lines = non-infected; solid lines = infected). B. Graphs summarizing data on average size of non-infected and infected cells in infections of SupT1.CIITA, MOLT-3 and Jurkat E6 with HHV-6B strain Z29 (all *n* = 3) and non-infected HSB-2 and HSB-2 cells infected with HHV-6A strain GS (*n* = 17). (PDF 6263 kb)

Additional file 3: ROC curves for size data analyzed as percentage of cells above the cutoff at 4 dpi (A) and 7 dpi (B). (PDF 197 kb)

Additional file 4: Alternative approaches for measuring size of cells. SupT1 were infected with various doses of HHV-6B strain Z29 and data collected at 7 dpi. A. Data collected using the imaging-based Cellometer Auto T4 cell counter: size distribution of non-infected (light gray) and infected cultures (dark gray). B. Data collected in a LSRII flow cytometer: forward-scatter area histograms of non-infected (light gray) and infected cultures (dark gray). (PDF 423 kb)

## Abbreviations

ANOVA: analysis of variance
AUC: area under the curve
cDNA: complementary DNA
dpi: days post-infection
FSA: forward scatter area
gB, gH, gQ: glycoprotein B, H, Q
HHV-6A: Human herpesvirus 6A
HHV-6B: Human herpesvirus 6B
HI: heat-inactivated
IFA: immunofluorescence microscopy
MFI: median fluorescence intensity
PCR: polymerase chain reaction
qPCR: quantitative (real-time) PCR
ROC: receiver operating characteristic
RT-PCR: reverse-transcription-PCR
SEM: scanning electron microscopy
SSA: side scatter area
TCID_50_: tissue culture infectious dose
TEM: transmission electron microscopy
UVI: inactivation by ultraviolet radiation
