# Supplementary figures and images for: Evaluation of a method to measure HHV-6B infection in vitro based on cell size
Source: Virol J. 2018 Jan 5;15:4. doi: 10.1186/s12985-017-0917-z (PMC5755443; doi:10.1186/s12985-017-0917-z)

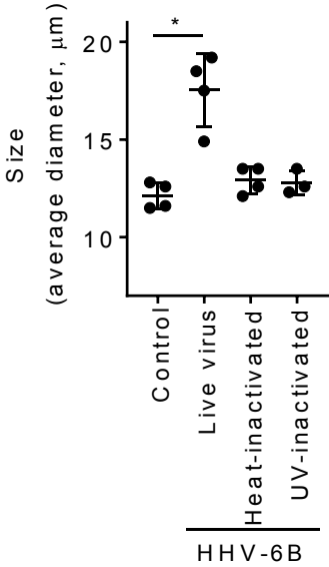

Supplement: Supplementary file 1 — Effect of heat-inactivation and UV-inactivation of HHV-6B in the average size of cells. Control: non-infected SupT1; HHV-6B: live, heat-inactivated, UV-inactivated. Statistical significant differences observed only between control and live virus. (PDF 76 kb) [file 12985_2017_917_MOESM1_ESM.pdf]

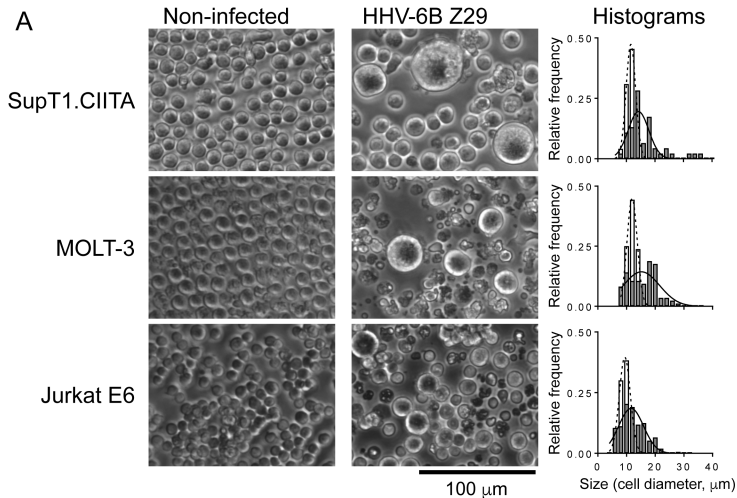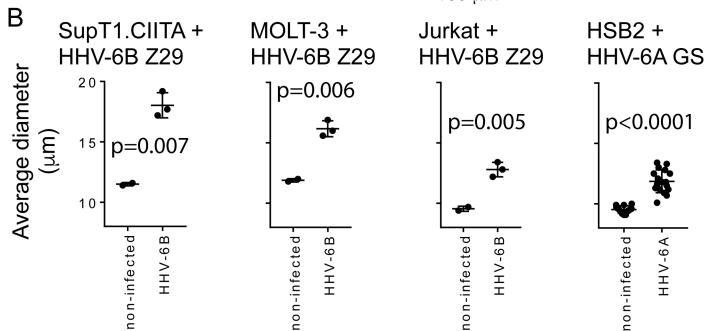

Supplement: Supplementary file 2 — A. Phase-contrast microscopy images (20×) of SupT1.CIITA, MOLT-3 and Jurkat E6 cells non-infected or infected with HHV-6B strain Z29 (4 dpi); the right panel shows size histograms of non-infected cells (clear bars) and infected cells (gray bars) for each of the aforementioned cell lines; also shown are the non-linear fits for each sample (dashed lines = non-infected; solid lines = infected). B. Graphs summarizing data on average size of non-infected and infected cells in infections of SupT1.CIITA, MOLT-3 and Jurkat E6 with HHV-6B strain Z29 (all n = 3) and non-infected HSB-2 and HSB-2 cells infected with HHV-6A strain GS (n = 17). (PDF 6263 kb) [file 12985_2017_917_MOESM2_ESM.pdf]

**A**

4 dpi

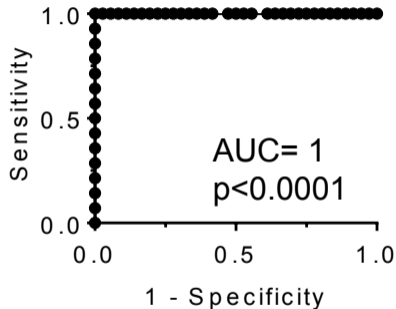

Cutoff 43.4%  
(100% sensitivity and specificity)

**B**

7 dpi

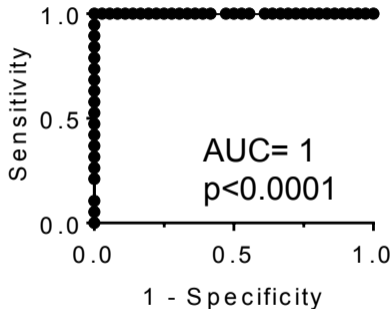

Cutoff 42.1%  
(100% sensitivity and specificity)

Supplement: Supplementary file 3 — ROC curves for size data analyzed as percentage of cells above the cutoff at 4 dpi (A) and 7 dpi (B). (PDF 197 kb) [file 12985_2017_917_MOESM3_ESM.pdf]

# Virus dose

Imaging-  
based cell  
counter  
(Cellometer  
Auto T4)

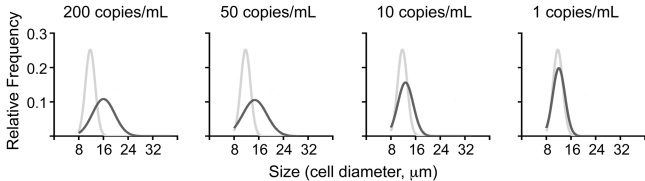

Flow  
Cytometer  
(BD  
Biosciences  
LRSII)

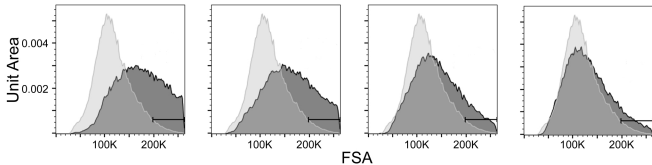

Supplement: Supplementary file 4 — Alternative approaches for measuring size of cells. SupT1 were infected with various doses of HHV-6B strain Z29 and data collected at 7 dpi. A. Data collected using the imaging-based Cellometer Auto T4 cell counter: size distribution of non-infected (light gray) and infected cultures (dark gray). B. Data collected in a LSRII flow cytometer: forward-scatter area histograms of non-infected (light gray) and infected cultures (dark gray). (PDF 423 kb) [file 12985_2017_917_MOESM4_ESM.pdf]
